# Dallas Meeting Texas State Medical Association

**Published:** 1886-05

**Authors:** 


DANIEL'S

### Medical ★ Journal,

PUBLISHED MONTHLY AT

AUSTIN, TEXAS.

F. E. DANIEL, M. D., Editor and Publisher

Subscription Price: Two Dollars per Annum in Advance.

Papers for publication in this Journal should be in the hands of the Editor by the 10th of the month preceding the month in which it is wished to appear; they must be original,—never having appeared in print elsewhere, and must be contributed exclusively to this Journal.

Contributions to the department of Original Articles are cordially invited from the professiom of Texas, expecially. What is most desired is Clinical reports of cases, essays and short practical articles on any medical topic; also solicited reports of proceedings of societies, clubs, etc., and items of medical news of general interest to the profession, such as notices of appointments of physicians, removals, changes, marriages, deaths, etc.

### Editorial.

### DALLAS MEETING OF THE TEXAS STATE MEDICAL ASSOCIATION.

Amongst our earliest obstetrical experiences, we remember one dark night, having been sent for in haste; arriving, we were saluted at the door by an old woman, with "the-trouble's-over-the-child's-born,-and-his-name-is-Ant'ny."

The long looked-for, and much talked of eighteenth annual convention of the Texas State Medical Association has passed into history. It was one of the largest, if not the largest gathering of medical men, ever held in Texas. The simultaneous assembling of the Texas State Pharmaceutical Association gave additional interest and eclat to the occasion; and we dare say, those four days will mark an era in the life, and will be a red-letter day in the calendar of many an "M. D."

In point of numbers, chiefly, and in the percentage of prominent, well-known physicians present, was it one of the most successful meetings,—all things being considered. In all, there were about 400 physicians in attendance; some going, some coming, there were perhaps not over 300 medical men present in the hall at any one time. At the State Pharmaceutical Association there were about 100 delegates.

Socially, it was a grand occasion, a most delightful reunion of physicians from all parts of the state; old, young and middle-aged,—fat, lean, big, little and medium-sized physicians; doctors with gold rimed-specs,ditto without; ditto with gold-headed canes, ditto without; physicians of all degrees of "regularity," from the ultra orthodox to the barely-passable-by-the-skin-of-the-teeth; and it is estimated by competent observers, and corroborated by the "oldest citizen," that there has not been in Dallas, the same amount of hand-shaking, and of introductions, since the first train on the "Central" came through from Galveston. Much credit is due the committee of arrangement, for the admirable programme they had arranged for the meeting; (it was no fault of theirs, that it was not better carried out) and for the splendid reception and entertainment of visitors. Their hospitality, and that of citizens generally, extended to the association, has endeared Dallas to every one present, and will long be remembered. We dare say, all left feeling glad they came.

Financially, too, the meeting may be regarded as eminently successful; inasmuch as the exchequer was replenished and made plethoric by the initiation fees and dues of nearly 100 new members; and old members, dropped for non-payment, reinstated; together with the customary anual *ante* of "all hands and the cook."

Estimated in the light of the medical work done—as a scientific meeting, we are constrained to say it was far short of some of its predecessors. This was the result of a combination of circumstances; for there was a predominating element of educated and experienced members present. We say this in no fault-finding spirit; far from it; but as the JOURNAL, the mission of which is the organization of the profession, is the avowed and acknowledged champion of its best interests, ever striving to elevate and maintain its dignity and honor, it is our duty to criticize the meeting, and to point out the errors, and the causes which operated to bring about a partial failure of the prime object for which the association assembled—the promotion of medical science. This we hope to do in a spirit of fairness and candor; and we do so in order that those errors may not be repeated ; and thus insure at our next meeting, a more satisfactory outcome, scientifically.

The membership is too large to longer conduct the meetings as heretofore, in a body; the body is unwieldy. Time was not economized, as it should have been; but was squandered often, in useless talk,—talk on subjects, at times, totally irrelevant, and out of order. It were a species of false modesty to deny that there is a chronic disposition to prolixity on the part of a number of members;—and some apparently do not consider what they shall say, but they *say* all the same, and keep on saying. We have felt at times, like we suppose the man felt who advertised for "an able-bodied man to hold his wife's tongue."

There are nine sections, and nine chairmen of sections;—and in each section there were presented from two to ten papers. (This is somewhat like the man who "went to St Ives," and "met the man with seven-wives," each wife having "seven sacks" etc., —excuse the digression.) Each chairman is expected,—required to read an address or report; and most of them, actuated, doubtless, by a commendable zeal, make the mistake of making it too long. This remark applies also to the average writer of a paper.

Again, ambition,—that inherent and animating principle of the human mind,—ambition to be seen and heard—appears to be epidemic, so to speak, amongst the members of the Association. We say *this* in no spirit of captiousness, for we have done much to induce, especially younger members, to write, and to take part in medical discussions; but, there is the trouble; everything else was talked of *but* medicine. In this way the time, which should have been devoted to reading and discussing papers, was taken up.

We mean to say, in brief, that there were some fifty papers contributed in all, and long ones; and it was not possible, in the nature of things, to get through with reading them. We may say,—indeed we have the authority of the secretary for saying, the majority of papers prepared, were *not* read ! there was no time to read them; because so much time was consumed in discussing the proposition to reduce the yearly dues; the proposition to vote a small pittance to the men who worked all of July and August (90 in the shade) to produce the volume of transactions which has challenged the admiration and envy of every State Medical Association in America, and which has elicited the warmest compliments from the Medical Press, and which moreover, has done much to add to the reputation of the Texas Association; in debating whether or not our delegates should go to St. Louis instructed etc., and in similar topics. However, the papers will appear in the published transactions; and that must be the balm to the wounded, crushed souls, who came away loaded as they went. If then, there was not time to read the papers which had been prepared, there was still less time to discuss such as were read. We heard it remarked by a number of physicans that they had not heard one word of discussion on any medical topic; and one member of a progressive local society, a distinguished physician, said he learned more from each meeting of his County Society than he had learned during the entire session of the State Association.

The time has come when the association must work in sections, or no progress will be made in medicine, however good time, socially, delegates may have. The forenoon should be given up to general sessions, in which the various features other than scientific, may be discussed and disposed of; and the afternoons to section work, as is the rule in the National Association; but, as the sections are too small to devote a hall and an afternoon to each, it is suggested by our zealous and capable secretary, and the suggestion is a good one, that there be three divisions; the first consisting of the sections of practice of medicine, and surgery; the second of obstetrics and disease of children, and gynæcology; and the third of all the remaining sections, which are the smallest; to-wit: State medicine, dermatology, electro-therapeutics and ophthalmology, ect. thus clubbing two or more sections together and giving a hall and an afternoon to each division. We suggest also, when there is press of work, as at Dallas, there might be an evening session, at least on those evenings not occupied with president's and essayist's addresses; say from 8 to 10 oclock; ten o'clock being early enough for "the ball" if ball there must be.

Another cause that operated to bring about confusion and loss of time was want of thorough acquaintance on the part of those who presided at different times—with parliamentary rules, etc. It is a humiliating spectacle to see some old stager respectfully call the attention of the chairman who may be presiding at the time, to the fact that such and such a thing "is not debatable," or to remind him that "a substitute has precedence over the resolution and must be disposed of first." The president and all the vice presidents, as well as the chairmen of sections, if they are not familiar with parliamentary rules, should by all means study them; and it is to be hoped the defects in this respect, so glaringly exhibited at Dallas, will not be repeated at Austin.

Take it all in all, the meeting was an occasion long to be remembered. It is another stone laid in the foundation, on which we hope to see erected a superstructure of surpassing grandeur. A united and purified profession—should and will command the respectful ear of the general assembly. We look to see a future board of medical advisors respectfully asked what measure they would suggest for the better protection of the public health; what sanitary laws for the prevention of epizootic diseases. We hope to see under the auspices of the Texas State Association, a grand medical department of the grand State University arise,—a diploma from whose walls—bearing the lone star, shall serve as a passport to the highest social and professional privileges—and which will command respect wherever the science of medicine has a foot hold on the civilized globe; and also an intelligent Board of Health with ample power. Then, some day, disease will be practically banished from our shores; and the mission of the physician will be to prevent and not to cure. Then, from the dome of the proudest capital in the world, the Goddess Hygiea will proclaim "health to the nations."

We may not hope to see this glorious picture realized; but our children's children—those who come after us will see, appreciate and profit by the labors and sacrifices of the medical men of this day. They will contemplate the realization of these aspirations and will rise up and call them blessed. They will look upon the record now being made, with reverential awe, and will bless the Texas State Medical Association as the source—the *protoplasm* whence sprung such grand development. So mote it be.

